# Carbon dots as a versatile tool to monitor insulin aggregation

**DOI:** 10.1007/s00216-023-04585-y

**Published:** 2023-02-20

**Authors:** Gabriele Antonio Zingale, Alessia Distefano, Irene Pandino, Nunzio Tuccitto, Valentina Oliveri, Massimiliano Gaeta, Alessandro D’Urso, Alfio Arcoria, Giuseppe Grasso

**Affiliations:** 1 IRCCS-Fondazione Bietti, Rome, Italy; 2 Chemical Sciences Department, University of Catania, Viale Andrea Doria 6, 95125 Catania, Italy

**Keywords:** Quantum dots, Surface chemistry, Covalent bond, Spectroscopy, Diabetes, Aggregation

## Abstract

The possibility to monitor peptide and protein aggregation is of paramount importance in the so-called conformational diseases, as the understanding of many physiological pathways, as well as pathological processes involved in the development of such diseases, depends very much on the actual possibility to monitor biomolecule oligomeric distribution and aggregation. In this work, we report a novel experimental method to monitor protein aggregation, based on the change of the fluorescent properties of carbon dots upon protein binding. The results obtained in the case of insulin with this newly proposed experimental approach are compared with those obtained with other common experimental techniques normally used for the same purpose (circular dichroism, DLS, PICUP and ThT fluorescence). The greatest advantage of the hereby presented methodology over all the other experimental methods considered is the possibility to monitor the initial stages of insulin aggregation under the different experimental conditions sampled and the absence of possible disturbances and/or molecular probes during the aggregation process.

**Graphical abstract:**

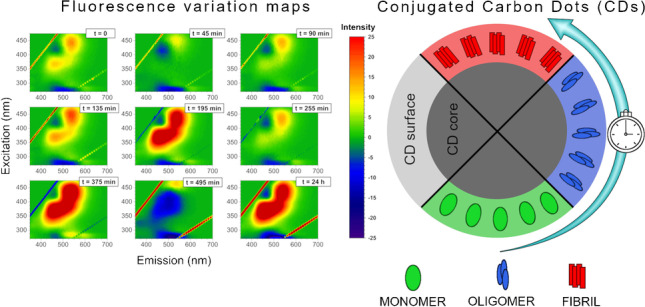

**Supplementary Information:**

The online version contains supplementary material available at 10.1007/s00216-023-04585-y.

## Introduction

Correct conformation and folding are of paramount importance for protein functioning, as development of pathological states and diseases, which typically affect nervous tissues (e.g. brain, retina), has been widely associated with protein misfolding and aggregation [1–3]. For this reason, bioanalytical tools, often based on fluorescence techniques, useful for monitoring protein aggregation [4], as well as therapeutic approaches based on inhibiting the latter [5, 6], have been widely investigated and proposed. Insulin aggregation has been widely studied with a variety of experimental techniques, ranging from capillary electrophoresis [7] to small-angle X-ray [8], as it is deposited in arterial walls of type II diabetes patients [9]. Molecules with potential theranostic value, which are capable to inhibit and monitor insulin aggregation, have also been recently proposed [10, 11], and the effect that experimental conditions and metal ions have on insulin aggregation has been deeply studied [12–16]. In the literature, several complementary techniques are available to investigate on protein aggregation. For example, SEC, AF4, PICUP and AUC have all been applied to investigate various proteins’ oligomerization and fibrillation [17–20]. Although each different bioanalytical approach offers specific advantages, it is important to highlight that usually particular experimental conditions must be taken into account to avoid problems and artefacts [19, 21].

In this scenario, carbon dots (CD) have also been proposed as possible insulin aggregation inhibitors, speculating that the observed inhibiting effect is likely due to the interaction between CD and insulin before elongation [22]. It has also been reported that it is possible to modulate the CD inhibiting properties by changing the chemical groups at the CD surface [23]. However, in all the previous studies, CD have never been used to monitor protein aggregation, as their fluorescent properties depend on the covalent chemical modifications of their surface, being unaffected by other molecules loosely present in solution. In a recent work, we have already demonstrated the possibility to apply the commonly used amine coupling reaction to covalently bind peptides to the CD surface, to being able to distinguish isomeric forms by the change in the CD fluorescence maps [24]. Here, we have investigated the possibility to follow protein aggregation by measuring the variations of the fluorescence maps of covalently attached insulin molecules at different stages of oligomerization to CD. It is widely reported that a three-stage mechanism consisting of protein misfolding, nucleation, and fibril elongation occurs during insulin aggregation [25] and most of the experimental techniques commonly used to monitor protein aggregation are blind to the initial stages of the process, being able to detect only β-sheet formation [26]. Here, we report on the possibility to monitor the initial stages of insulin aggregation at the different experimental conditions sampled, demonstrating the advantages offered by the use of CD for protein aggregation over some of the most common experimental techniques commonly used for the same purpose. An insight into insulin fibrillation in the presence and in the absence of zinc ions is also given. Given that CD are also strong inhibitors for insulin aggregation, our findings indicate the possibility to use such nanoparticles as potential theranostic agents in amyloidogenic diseases.

## Materials and methods

### Carbon dots’ preparation

**Scheme 1:**
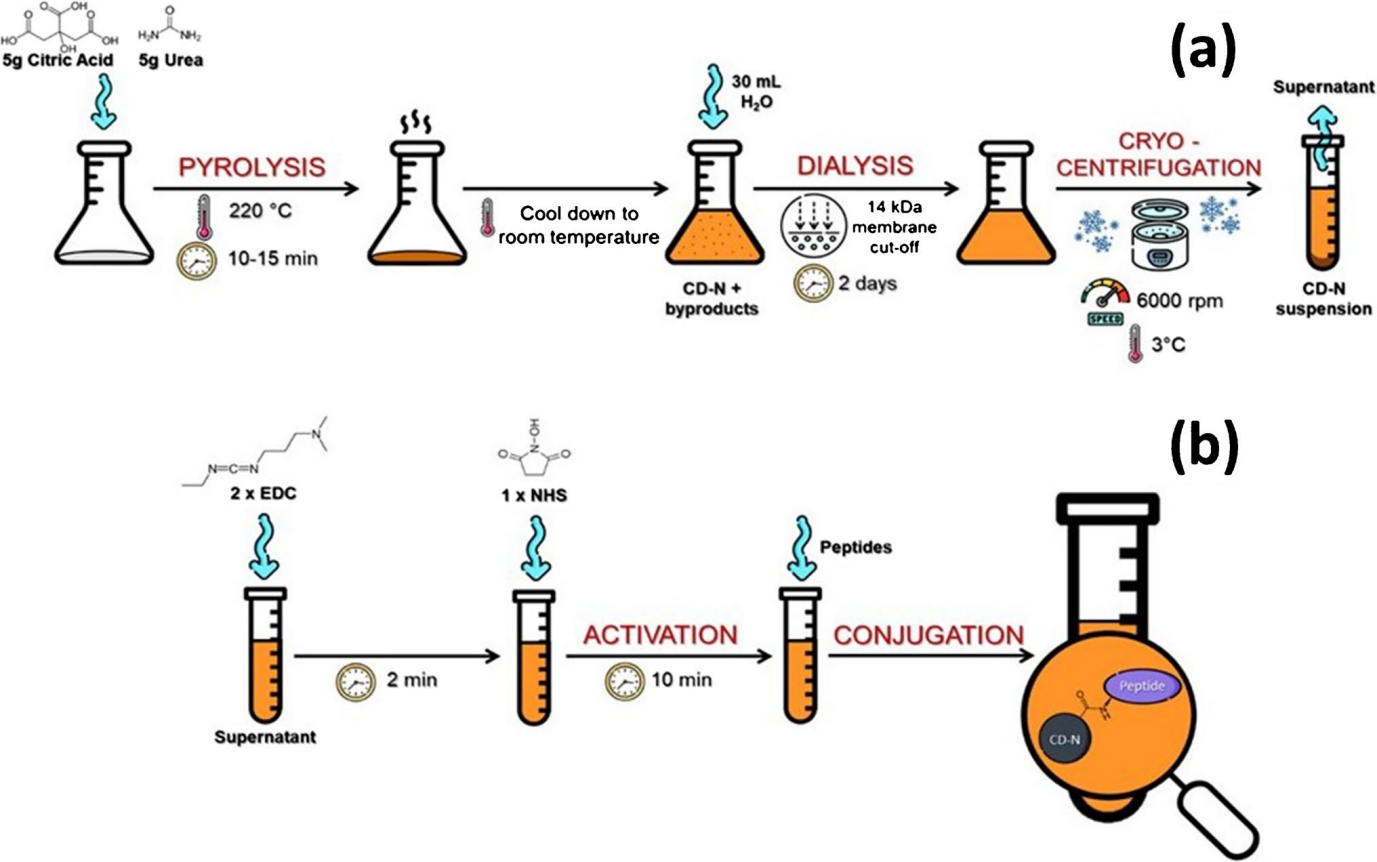
(**a**) Synthesis of CD–N and (**b**) their use to monitor insulin aggregation

**Fig. 1:**
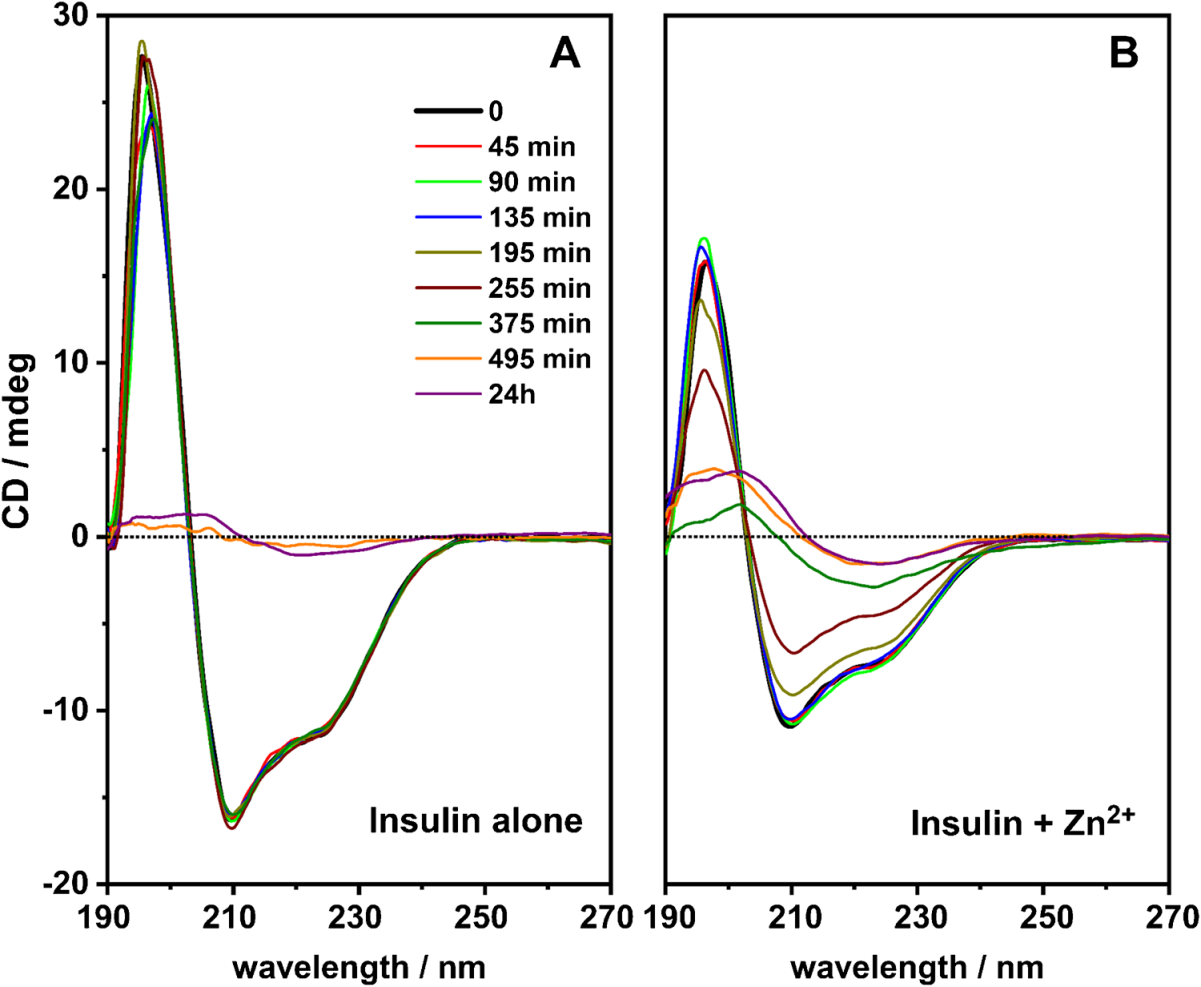
CD spectra during the incubation of insulin alone (panel **A**) and in the presence of zinc ions (panel **B**). In all spectra, the insulin concentration is 3 µM and the pH = 3.5. In panel B, the ratio [insulin]/[Zn^2+^] is 1:1

**Fig. 2:**
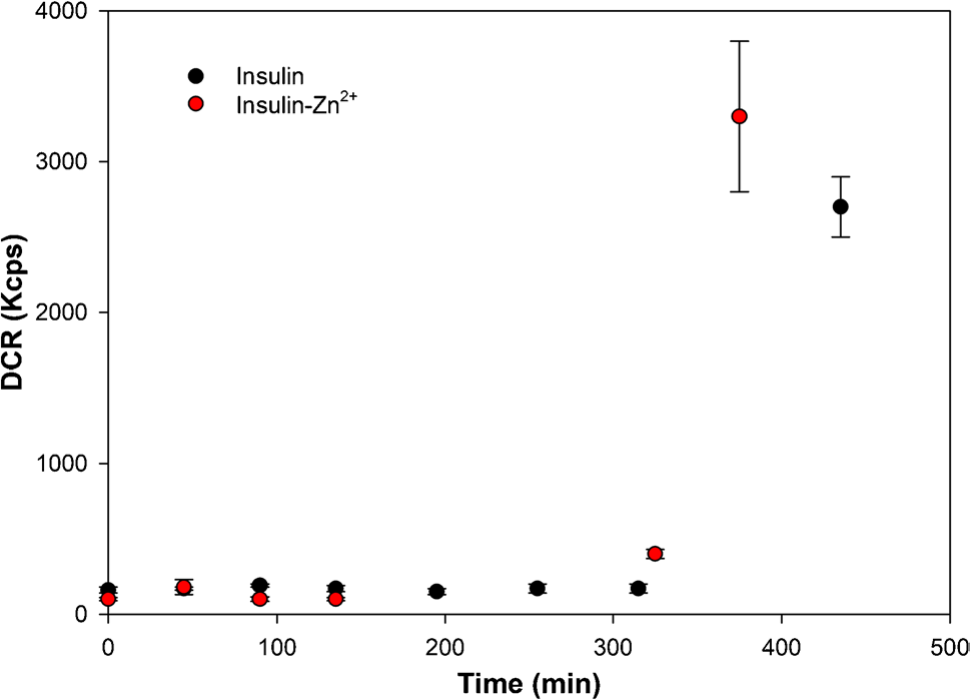
The scattering intensity, denoted by the derived count rate (DCR), of insulin samples vs time in the presence and in the absence of Zn^2+^. Error bars show standard deviations

**Fig. 3:**
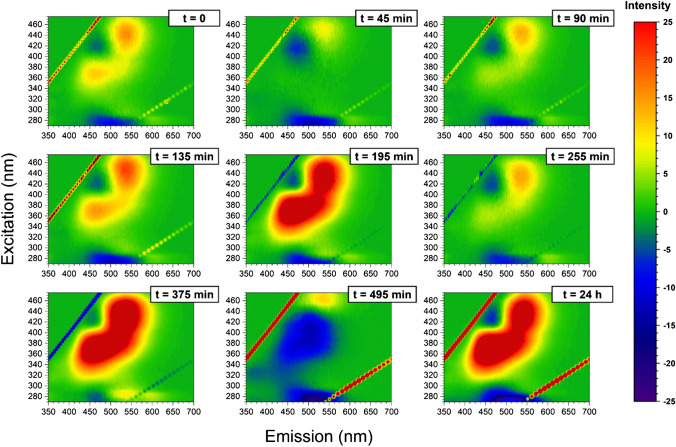
3D fluorescence variation maps of 0.0096 mg/mL CD solution conjugated to insulin (20 μM) at each time point of the incubation (1-mM solution of insulin at 60 °C)

Carbon dots were prepared by thermal decomposition of citric acid (from Merck, Italy) as previously reported (see Scheme 1a) [24]. Briefly, in order to obtain CD enriched in amino groups on the surface, 2.5 g of citric acid and 2.5 g of urea (from Merck, Italy) were weighed and placed on a hot plate at approximately 220 °C. The pyrolysis process lasts for 10–15 min, until a kind of yellowish caramel is obtained. This is cooled to room temperature, and then, 50 mL of ultrapure water obtained from PURELAB Flex 3 system—Elga Veolia company is added to it. The result is a suspension of CD with other by-products of thermal decomposition. In order to obtain a suspension as clean as possible, a dialysis process is continued using a membrane with a cutoff of 14 kDa (from Merck, Italy). Dialysis ends when the dialysis water no longer fluoresces, i.e. after about 2 days. The resulting suspension is then subjected to cryo-centrifugation at 3 °C, at 6000 rpm for 1 h. The supernatant corresponds to the final suspension, one desired for subsequent characterization and experiments. With this thermal decomposition method, a typical CD concentration of about 2 mg/ml is obtained. Characterization of the obtained CD is reported in the Supplementary Materials (Fig. 1S, Fig. 2S and Fig. 3S).

### Insulin incubation

For the fibrillation of insulin alone, 2.5 mL of a 1-mM insulin solution (from Laborchimica Srl, Italy, prepared dissolving 14.52 mg in 2.5 mL) in PBS buffer pH 1.6 (137 mM NaCl, 2.7 mM KCl, HCl and 10 mM phosphate buffer from Merck, Italy) was filtered through a 0.2-μm pore nylon filter and then incubated at 60 °C. Briefly, at different time points, an aliquot of the solution was diluted and analysed with the techniques described in the following text.

For the fibrillation of insulin in the presence of zinc ions, 2.0 mL of a 1-mM insulin and 1-mM ZnSO_4_ (from Merck, Italy) solution in PBS buffer at pH 1.6 was filtered through a 0.2-μm pore nylon filter and then incubated at 60 °C. Briefly, at each time point, an aliquot of the solution was diluted and analysed with the techniques described as follows.

### Circular dichroism

The insulin work solutions were prepared at room conditions by withdrawing the proper amount of insulin stock solution (= 1 mM from Laborchimica Srl, Italy), kept under fibrillation at 60 °C, in 2 mL of diluted PBS buffer from Merck, Italy (dilution factor = 1:100) in order to get a final insulin concentration of 3 µM. In particular, the PBS buffer (pH = 1.6, adjusted by HCl conc.) was diluted by ultrapure water obtained from PURELAB Flex 3 system—Elga Veolia company. In another experimental set, an identical insulin stock solution (= 1 mM) was incubated at 60 °C in the presence of zinc sulfate salt, ZnSO_4_ ([Zn^2+^] = 1 mM). The corresponding work solutions were prepared as aforementioned. Finally, all as-prepared insulin solutions exhibited pH values around 3.5.

The monitored incubation’s point times were as follows: 0 min–45 min–90 min–135 min–195 min–255 min–375 min–495 min–24 h.

The JASCO J-715 spectropolarimeter equipped with a 1-cm path-length cell was used for the circular dichroism investigations at room temperature. A 1-cm quartz cuvette was used for all spectroscopic measurements.

### Dynamic light scattering

Buffer (PBS, from Merck, Italy, pH 1.6) and insulin solutions from Laborchimica Srl, Italy, were filtered through a 0.2-μm filter before the beginning of each aggregation experiment. Aliquots from the aggregating samples at different time points were diluted with the buffer (pH 1.6) to obtain an insulin concentration of 100 μM for DLS measurements. The measurements were performed at 25 °C with a Zetasizer Nano ZS (Malvern Instruments Ltd., UK) instrument equipped with a He–Ne laser. DLS measurements were performed on disposable microcuvettes by using optimal measurement times and laser attenuation settings. The angle of the scattering light used for size determination was 173°. Sample analysis was based on water viscosity (0.88 mPa s) and refractive index (1.33) at 25 °C. All samples were measured 4–5 times with 10–15 accumulated scans.

### Photo-induced cross-linking of unmodified proteins (PICUP) and SDS-PAGE

A solution of 90 mM APS (Merck, Italy) in 10-mM PBS and a solution of 4.5-mM Ru(bpy) (Merck, Italy) in 10-mM PBS were prepared. These concentrations were chosen in order to obtain the following molar ratios: insulin:Ru(bpy):APS = 1:2:40. The insulin solution was incubated at 60 °C and 150-µL aliquots were taken at different time points: 0 min (time point 1)–45 min (time point 2)–90 min (time point 3)–135 min (time point 4)–195 min (time point 5)–255 min (time point 6)–375 min (time point 7)–495 min (time point 8)–24 h (time point 9). The irradiation time chosen to study insulin fibrillation at different incubation times was 1.5 s. After the irradiation, we proceeded with the addition of the reducing buffer. Reducing buffer is a 5% solution of 2-mercaptoethanol (Merck, Italy) in sample buffer, which consists of the following: 3.55 mL H_2_O, 1.25 mL 0.5-M Tris–HCl buffer pH 6.8, 2.5 mL of glycerol, 2 mL of SDS 10% (w/v) and 0.2 mL of bromophenol blue 0.5% (w/v) (all reagents were purchased from Merck, Italy). The volume of reducing buffer must be in a 1:1 ratio with the insulin solution obtained at the end of the PICUP experiment. Finally, about 100 µL of this solution is transferred into an Eppendorf and this is placed in a water bath at a temperature of about 95 °C for 5 min in the dark.

The covalent oligomers obtained by the PICUP experiment are separated by discontinuous sodium dodecyl sulfate–polyacrylamide gel electrophoresis (SDS-PAGE). In this case, a 5% stacking gel and a 17% resolving gel were used. After the electrophoretic run, the peptide bands present in the gel were stained using a Coomassie Blue R250 solution (Merck, Italy). Approximately 45 min is needed for a complete colouring. Subsequently, we proceeded with its decolourization. In this phase, the excess dye is removed with the aid of two solutions called decolorizing solution A (500 mL of methanol, 100 mL of glacial acetic acid and 1 L of deionized water) and decolorizing solution B (70 mL of methanol, 100 mL of glacial acetic acid and 1 L of deionized water). In decolorizing solution A, the gel must be left for about 1 h, while in decolorizing solution B the time necessary to obtain clearly visible bands. After destaining, the gel was photographed and analysed using ImageJ software.

### Carbon dots-insulin conjugation and fluorescence method

Carbon dots’ activation was carried out, as previously described (see Scheme 1b) [24]. Briefly, 10 min before the given time point, EDC (1-ethyl-3-(3-dimethylaminopropyl)-carbodiimide hydrochloride, from Merck, Italy) and NHS (N-hydroxy succinimide, from Merck, Italy) were added as powder to the CD solution (50-mM and 25-mM final concentration, respectively, to have a molar ratio 2:1 EDC:NHS). NHS was added 2 min after the addition of EDC.

Once the activation of CD surface was reached, an aliquot of the insulin-incubated solution was added to the activated CD solution (exactly 10 min later), in order to have a sample with a final nominal concentration of insulin of 20 µM. All the fluorescence measurements were performed using a Varian Cary Eclipse fluorescence spectrophotometer. All the 3D emission maps were acquired using a 3-mL quartz cuvette filled with 2.5 mL of sample. The instrument was set at the following wavelength ranges, which were found to be the optimal choice in terms of resolution and time of acquisition (~ 13 min for each fluorescence map): excitation range: 270–475 nm; emission range: 350–700 nm; excitation increment: 5 nm; excitation/emission slit width: 3 nm/3 nm; sampling interval: 2 nm. A preliminary fluorescence variation emission map of a 0.0096 mg/mL CD-activated solution (in PBS pH 1.6) without insulin was acquired under the conditions cited above. The fluorescence variation is given by the difference between the activated CD solution and the conjugated insulin-CD solution. The CD suspensions used in all experiments have the same concentration (0.0096 mg/mL). Evidence that the CD surface has been modified by the EDC/NHS conjugation has been already reported by us [24] and it is anyway demonstrated by the changes in the fluorescence variation maps obtained in the presence of insulin at the different times of incubation, as discussed in the next section. Without CD activation, we obtain a mixture of CD and peptides and not a conjugation between the two systems. In this case, if the insulin is incubated with non-activated CD, the fluorescence variation maps recorded remain exactly the same at the various times of incubation (data not shown). On the other hand, as demonstrated below, if the CD surface is activated with EDC/NHS, a strong interaction (conjugation) of insulin with the nanoparticles is obtained, resulting in clear variations in the fluorescence variation maps, depending on the insulin oligomeric/aggregated species present in solution at the specific time when the conjugation with the CD is carried out.

The Thioflavin T (ThT) 3D assay was performed taking an aliquot from the incubation solution and mixing it with a solution of ThT 20 μM in PBS at pH 1.6 (HCl addition) to reach a final concentration of insulin of 20 μM. At each time point, a 3D fluorescence emission map of the solution was acquired under the same conditions cited above.

## Results and discussion

Insulin was incubated as reported above in order to induce fibrillation. Many spectroscopic techniques can be used to detect the formation of insulin fibrillation [27–29]. Indeed, several conformational events accompany the insulin aggregation, establishing unique superstructural architectures [30, 31]. In this context, circular dichroism spectroscopy can represent a straight way to monitor the insulin conformational changes throughout the fibrillation phenomenon [32]. Insulin, in its native form, is predominantly twisted in an α-helix conformation. On the contrary, it progressively envelops into β-sheet fibrils during its aggregation [33, 34]. This gradual transition can be disclosed from the dichroic study over time of the corresponding incubated solution (Fig. 1). In fact, our circular dichroism investigations indicate that, at first, the insulin displays a clear α-helix conformation (Fig. 1—panel A, black curve) as denoted by a negative peak with a typical “dip” between two relative minimums at 222 nm (n → π* transition) and 208 nm (π* → π transition) [35]. Moreover, π* → π transitions of the peptides in a polypeptide chain all couple together. For instance, the negative signal at 208 nm is due to the polarization parallel to the helix axis, whereas the strong positive signal below 200 nm is ascribable to the polarization perpendicular to the helix axis. Moreover, the α-helix conformation is often larger in magnitude than that due to other motifs.

We did not observe significant conformational changes up to 6 h (Fig. 1—panel A). However, as of 6 h of incubation, the stock solution became opalescent, likely owing to the peptide aggregation and formation of abundant insulin fibrils. Such behaviour was also confirmed by the corresponding circular dichroism spectrum (Fig. 1—panel A, orange curve). Here, a typical β-sheet conformation appears, characterized by a negative band at about 218 nm and a positive band of comparable magnitude near 195 nm. At the end of incubation (time = 24 h), no further variations can be revealed (Fig. 1—panel A, purple curve). In addition, we decided to test the insulin aggregation in the presence of zinc ions, as the presence of the latter is reported to have a major effect on insulin oligomerization and aggregation state [13, 14, 16].

In this case, gradual conformational changes come into view over time (Fig. 1—panel B). In particular, the first incubation steps (until 2 h) suggest us a steady α-helix conformation (Fig. 1—panel B, black, red, green and blue curves). However, as of 2 h, insulin tends to progressively assume a β-sheet conformation. The transition towards the new conformation gets finished at the last parts of the incubation (time = 495 min) remaining unaltered after 24 h as well (Fig. 1—panel B, orange and purple curves).

Overall, circular dichroism spectroscopy turns out to be a powerful technique to inspect and verify the insulin conformational variations within the aggregative phenomena. However, it proves delicate to discern small conformational changes whether (i) the chromophores do not own a high molar extinction coefficient (e.g. insulin) and (ii) the chromophores’ electronic coupling is not efficient enough to produce a sharp cotton effect [36]. For instance, this latter can occur in the early stage of the insulin fibrillation. For that reason, additional tools are advisable to deeply unveil the insulin fibrillation, which is then a kinetically and thermodynamically driven process.

Dynamic light scattering (DLS) is also a technique used to monitor protein aggregation. In particular, DLS is often applied to observe changes in the hydrodynamic diameter (diameter of hydrated molecule assuming a perfect sphere) of proteins during the aggregation process [37, 38]. Moreover, the derived count rate (DCR) of scattered photons plotted versus time can give information on the kinetics of the aggregation. DCR depends on both the number of scatterers and their size [39]; therefore, an increase in the DCR values during insulin incubation indicates the formation of protein aggregates. DLS data of insulin in the absence and presence of zinc ions are shown in Fig. 2. The DCR of insulin alone remained relatively constant for about 5 h; then, it increased. DCR enhanced slightly faster in the presence of zinc ions. Our findings suggest that DLS can give information on the aggregation kinetics but does not allow us to follow the initial variations that occur during the aggregation process of insulin. Some information on the changes of the insulin oligomeric distribution on incubation time can be obtained with different techniques, more sensitive to small oligomers. As an example, we have applied the PICUP/SDS-PAGE approach for this purpose, as it has been already reported that such a method is sensitive also to detect small oligomers [20]. Although we have been able to monitor some changes in the distribution of monomer and dimer abundancies at different insulin incubation times (see Supplementary Materials, Fig. 4S, Fig. 5S and Fig. 6S), this method failed to give an insight on higher oligomeric species distribution.

**Fig. 4:**
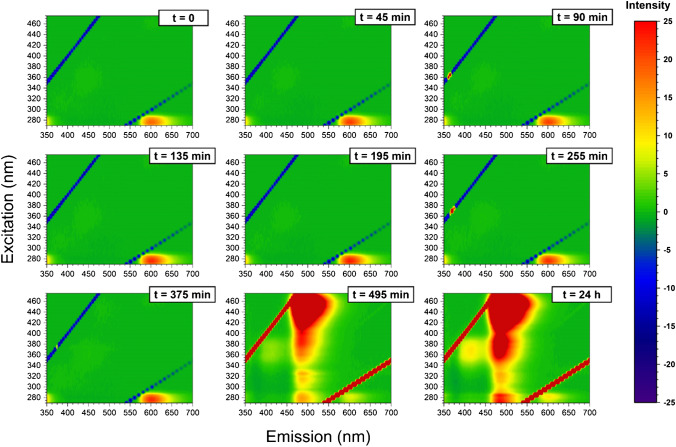
3D fluorescence variation maps of 20-μM ThT solution mixture with insulin (20 μM) at each time point of the incubation (1-mM solution of insulin at 60 °C)

**Fig. 5:**
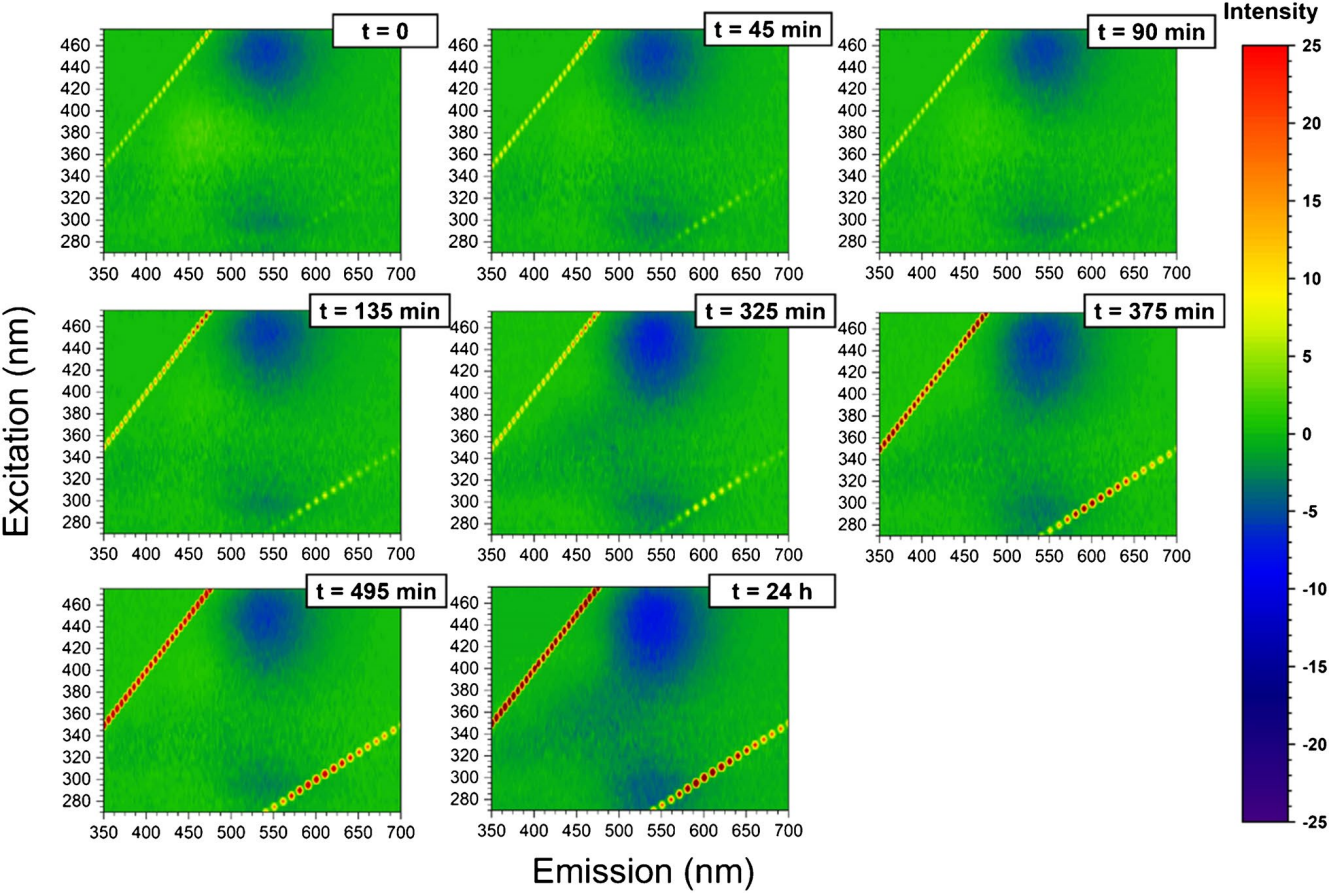
3D fluorescence variation maps of 0.0096 mg/mL CD solution conjugated to insulin (20 μM) in the presence of Zn.^2+^ (20 μM) at each time point of the incubation (1-mM solution of insulin and 1 mM of ZnSO_4_ at 60 °C)

**Fig. 6:**
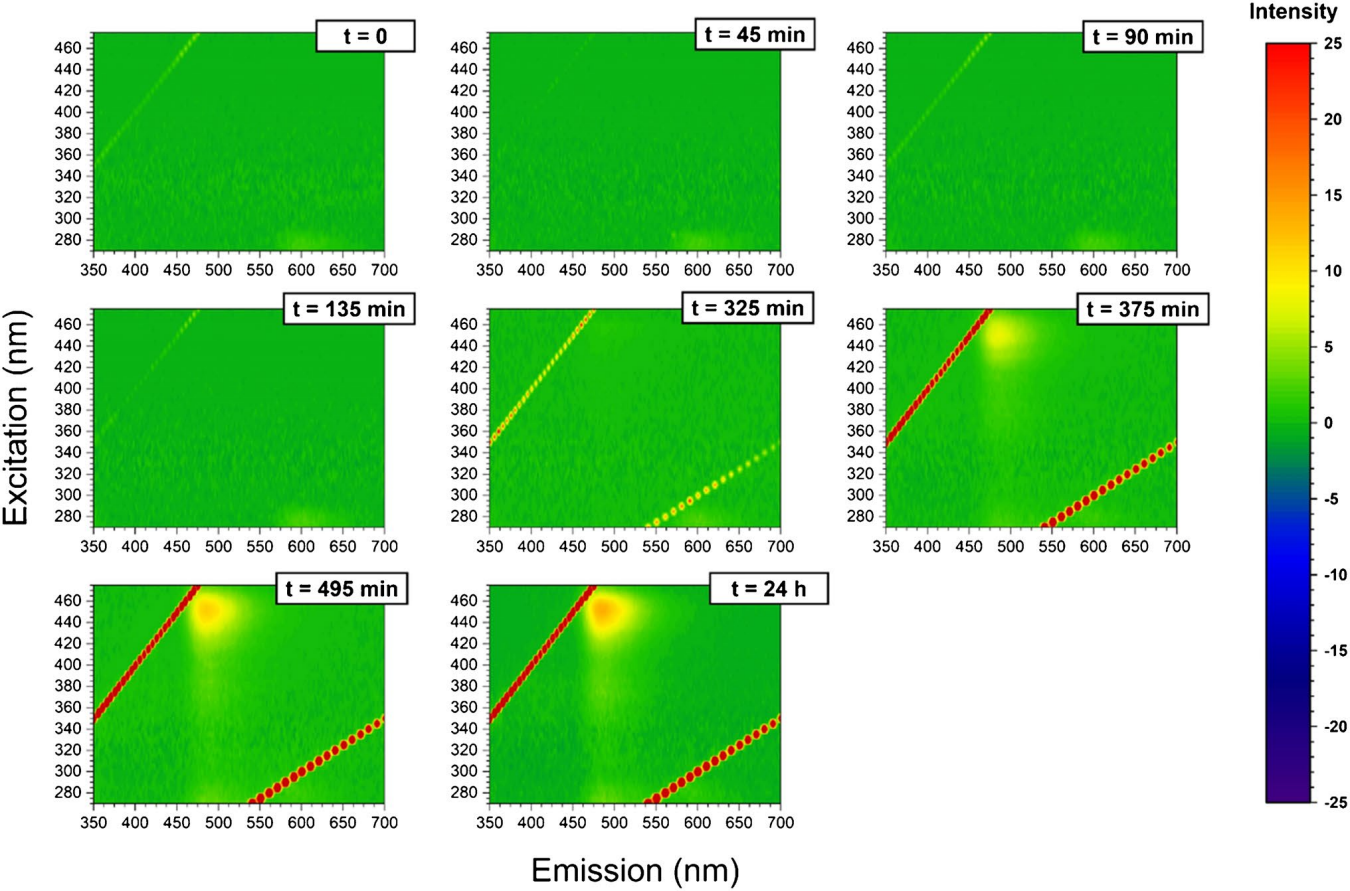
3D fluorescence variation maps of 20-μM ThT solution mixture with 20-μM insulin, at each time point of the incubation (1-mM insulin and 1-mM ZnSO_4_ solution at 60 °C)

It has been shown that emission/excitation map variations are a reliable and sensitive way of selectively detecting amino acids [40] and are extremely sensitive to the different orientation conformation of oligo peptides [24]. In Fig. 3, the 3D fluorescence variation maps of 0.0096 mg/mL CD covalently conjugated to insulin solution (20 μM) at each time point of incubation are reported. It is possible to see that, although the major changes in the map occur at time longer than 375 min, small but appreciable variations in the maps can be detected gradually at each time point. As a comparison, the same variation maps have been also obtained by using the ThT dye and the results are reported in Fig. 4. Although ThT is the most widely used tool to monitor amyloid formation, it is only able to detect amyloid-β sheets, whereas it is totally blind to insulin misfolding and/or oligomerization. On the contrary, the insulin covalent conjugation to CD alters the fluorescence properties of the latter, giving rise to a change in the variation maps that is dependent to every change of insulin conformation and/or oligomerization. Indeed, insulin conjugation occurs through the carboxy groups of its chains [24] and the exposure of the latter to the solvent, that is their steric availability for the coupling reaction, is directly dependent on the protein folding and oligomerization, rather than just on the formation of amyloid-β sheets. From Fig. 3, it is possible to see that the carboxy groups’ exposure to the solvent of insulin molecules is very similar for the three time points taken at 195 min, 375 min and 24 h, demonstrating that insulin conformational changes are occurring, in a gradual manner, well before the formation of fibrils detected by ThT after 375 min of incubation. Interestingly, insulin molecules seem to change their carboxy groups’ exposure to the solvent and, consequently, their steric arrangement on the CD surface inducing the different observed fluorescence, at about 195 min of incubation in our experimental conditions. These changes, possibly due to the formation of oligomeric species, are invisible to the ThT detection, sensitive only to amyloid fibrils. Of course, at all incubation times, there is an equilibrium between monomeric and oligomeric insulin species and we are only able to monitor the shift in the relative abundances of the species evolving. Indeed, as fibrils form from the monomeric insulin, insulin aggregation is inhibited by the presence of oligomers (hexamers above all) and we observe a fluorescent variation map at 255 min that is similar to the one observed for short times of incubation, demonstrating that, before the formation of prefibrillar aggregates, the disruption of oligomeric species and the formation of unfolded insulin occur. Similarly, at 495 min, the prefibrillar forms give the way to unfolded insulin, which then evolves towards the amyloid-β sheets detected also by ThT. Such explanation of the observed variation maps is also confirmed by the experiments carried out in the presence of zinc ions. Zinc is well known for stabilizing insulin hexamers and decreasing the hormone fibrillation [12, 13, 16]. In Fig. 5, the 3D fluorescence variation maps of 0.0096 mg/mL CD solution conjugated to insulin (20 μM) in the presence of Zn^2+^ at each time point of the incubation are reported and it is possible to see that they are very different from the ones reported in Fig. 3. In this case, fluorescence is quenched rather than increased, even at very long incubation times, and this finding can be explained only by assuming that, in the presence of the metal ions, different distributions of oligomeric species are present in comparison to insulin alone, so that the binding to the CD occurs in a different manner. Interestingly, a transient decrease of the fluorescence was also observed for insulin alone for 495 min of incubation, possibly indicating a transient abundance of specific oligomeric species, prodromal to fibrils, for this time point. As a comparison, in Fig. 6, the ThT results are also reported for the insulin-zinc solution and the only information that can be obtained in this case is that zinc diminishes the overall amount of insulin fibrillation, as already reported. However, a limited formation of β sheets occurs at smaller times of incubation (375 min), in accordance with the CD, PICUP/SDS-PAGE and DLS results reported above.

**Fig. 7:**
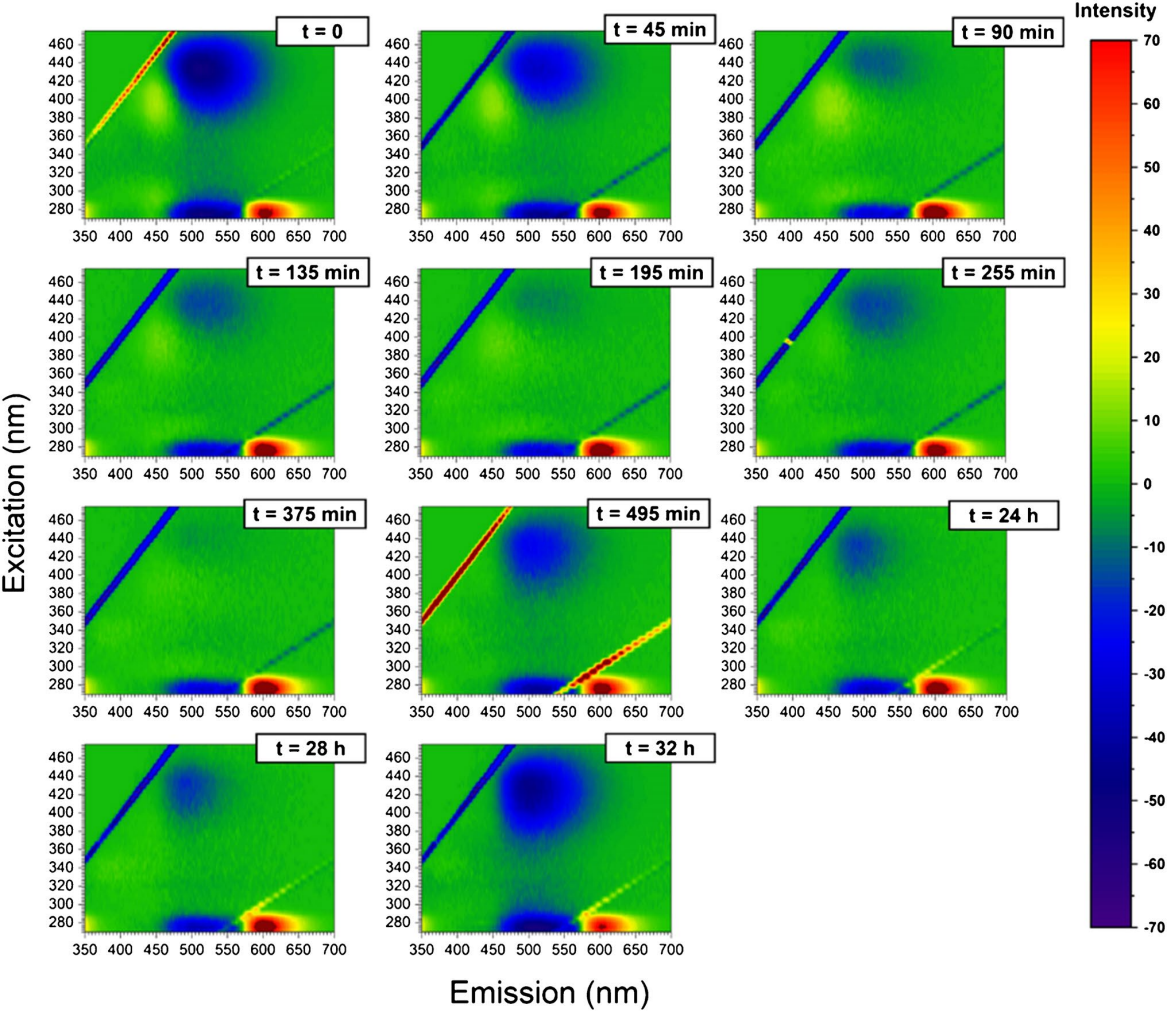
3D fluorescence variation maps of solutions (final composition: insulin 20 μM and CD 0.0096 mg/mL) sampled at the incubation times indicated (*T* = 60 °C), from a mother solution containing 1-mM insulin and 0.43 mg/mL of CD conjugated at time 0

Our results indicate that the CD fluorescence is only dependent on the chemical composition of the surface, while it is independent on whatever is occurring in the solution. The changes in the variation maps occur because at the different incubation times, the various insulin species present in the solution have a specific concentration equilibrium, which determines a unique overall carboxy groups’ exposure and, in turn, a unique insulin coverage of the CD surface. Indeed, CD are unresponsive to what is happening in the solution if insulin molecules are not covalently bound to the surface, as in this case the fluorescent variation maps remain unaltered at the various incubation times (data not shown). However, even if insulin molecules are conjugated to CD at the beginning of the incubation and then a solution aliquot is sampled at the different times of incubation, very little differences are recorded in the fluorescent variation maps. In Fig. 7, the results are reported and it is possible to see that the variation maps are very different from the ones reported in Fig. 3 and discussed above. Indeed, if insulin is covalently bound to the surface of the CD at the beginning of the incubation time, the fluorescence variation maps are not very responsive to the changes in insulin conformations and/or aggregation states, as expected. Indeed, in this case, the insulin molecules attached to the CD surface do not participate significantly to the aggregation process as the ones which are in solution, therefore inducing negligible changes in the fluorescent variation maps.

## Conclusions

A new experimental methodology to monitor protein fibrillation has been proposed and applied in the case of insulin aggregation. Particularly, CD have been used for the first time as a new tool to assess changes in insulin conformation and/or aggregation state in the presence and in the absence of zinc ions, and the results are compared with those obtained by other commonly used experimental techniques such as circular dichroism, PICUP/SDS-PAGE, DLS and ThT fluorescence. Particularly, although circular dichroism spectroscopy turns out to be a powerful technique to inspect and verify the insulin conformational variations within the aggregative phenomena, it proves delicate to discern oligomeric distribution. On the other hand, both DLS and ThT fluorescence prove to be very useful for the detection of amyloid fibrils, but are not capable to detect early-stage aggregation processes. PICUP/SDS-PAGE gave an insight on the monomers/dimers distribution, but failed to monitor the formation of higher insulin oligomeric and fibrillary species. For this reason, additional tools are advisable to deeply unveil the insulin aggregation, which is then a kinetically and thermodynamically driven process. It is clear that the newly proposed method allows the monitoring of early-stage aggregation states not visible by the other experimental approaches, providing a new application of CD to the field of protein conformation and aggregation. Indeed, the fluorescent properties of CD are very sensitive and responsive to the conformational and aggregation state of any protein which is covalently attached to the CD surface. Our results demonstrate that the newly designed method hereby proposed allows to take a snapshot of the aggregation state of the protein in solution at a particular time of incubation, only if the conjugation with the CD is carried out at that specific time and not at the beginning of the incubation. It is important to highlight that such experimental design also guarantees that the aggregation process of the protein under analysis is not perturbed in any way by the presence of molecular probes or surfaces (CD have been shown to delay insulin aggregation). Such major advantages over other commonly used techniques (ThT above all), together with the possibility to monitor early-stage aggregation states, give huge potentials to the presented method, which we will apply to other systems in the near future. An in-depth comparison of the potentiality of this newly proposed experimental approach with many other available bioanalytical techniques such as SEC, AUC or AF4 is also highly desirable in order to further assess all the advantages and flaws of our proposed approach.

## Supplementary Information

Below is the link to the electronic supplementary material.

Supplementary file1 (DOCX 1942 KB)
