# Supplementary material for: Carbon dots as a versatile tool to monitor insulin aggregation
Source: Anal Bioanal Chem. 2023 Feb 20;415(10):1829–40. doi: 10.1007/s00216-023-04585-y (PMC10049934; doi:10.1007/s00216-023-04585-y)
Supplement: Supplementary file 1 — Supplementary file1 (DOCX 1942 KB) [file 216_2023_4585_MOESM1_ESM.docx]

**Supplementary Materials**

**Carbon dots as a versatile tool to monitor insulin aggregation**

*Gabriele Antonio Zingale,^1^ Alessia Distefano,^2^ Irene Pandino,^2^ Nunzio Tuccitto,^2^ Valentina Oliveri,^2^ Massimiliano Gaeta,^2^ Alessandro D’Urso,^2^ Alfio Arcoria,^2^ Giuseppe Grasso^2*^*

*^1^ IRCCS-Fondazione Bietti, Rome, Italy*

*^2^ Chemical Sciences Department, University of Catania, Viale Andrea Doria 6, 95125, Catania, Italy*

**grassog@unict.it*

**Carbon Dots Characterization**

The typical elemental composition of CD–N were, C 62(±2) %; O 19(±2) %; N 16(±2) %; H 3(±2) %. The elemental analysis was performed by using the PerkinElmer 2400 Series II CHNS/O on dry samples. A typical FT-IR spectrum of CD–N is reported in Fig. 1S. The spectrum, was acquired with JascoFTIR, dispersing dried CD-N in KBr pellets. Spectrum shows the characteristic absorption peaks for the stretching vibration of C=O around 1711 cm^-1^, C-O bonds at 1205 cm^-1^, O-H at 3363 and 3033 cm^-1^, and C-H or O-H bending at 1395 cm^-1^, symmetric stretching vibration at 1570 cm^-1^ for a N-H bond, various characteristic peaks at 2940 and 2855 cm^-1^ related to CH_2_ asymmetric and symmetric stretching vibrations.

**Figure 1S**: FT-IR spectrum of CD-N

The UV-vis absorption spectrum (acquired with a Jasco 670 instrument) of aqueous CD-N solutions is shown in Figure 2S. The results are compatible with the straw-yellow coloration of the solutions and the excitation spectra reported in the main article.

**Figure 2S**: UV spectrum of CD-N

Atomic force microscopy (AFM) characterization was performed with a Nanoscope IIIA instrument (Digital Instruments). Figure 3S reports a typical image of CD-N deposited onto mica substrate by drop casting and subsequent water evaporation. The concentration of the solution was adjusted by diluting up to self-aggregation of particles was avoided. The most common dimension of the particles is around 7 nm.

**Figure 3S**: AFM micrography of CD-N deposited on to mica

**Photo-induced cross-linking of unmodified proteins (PICUP) and SDS-PAGE**

The image of the gel (Fig. 4S) was used to be processed and analyzed using ImageJ (a computer program for digital image processing), which allows to obtain the intensities of the peptide bands present in the gel.


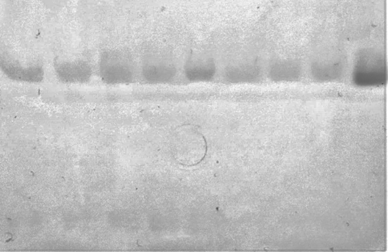


**Figure 4S**: Gel image processed using the ImageJ program.

Subsequently, the signal areas are integrated and numerical values ​​are extracted, from which, using Origin, a histogram is obtained. In Fig. 5S and 6S the histograms relative to the monomeric and dimeric species at different incubation times are reported, respectively.


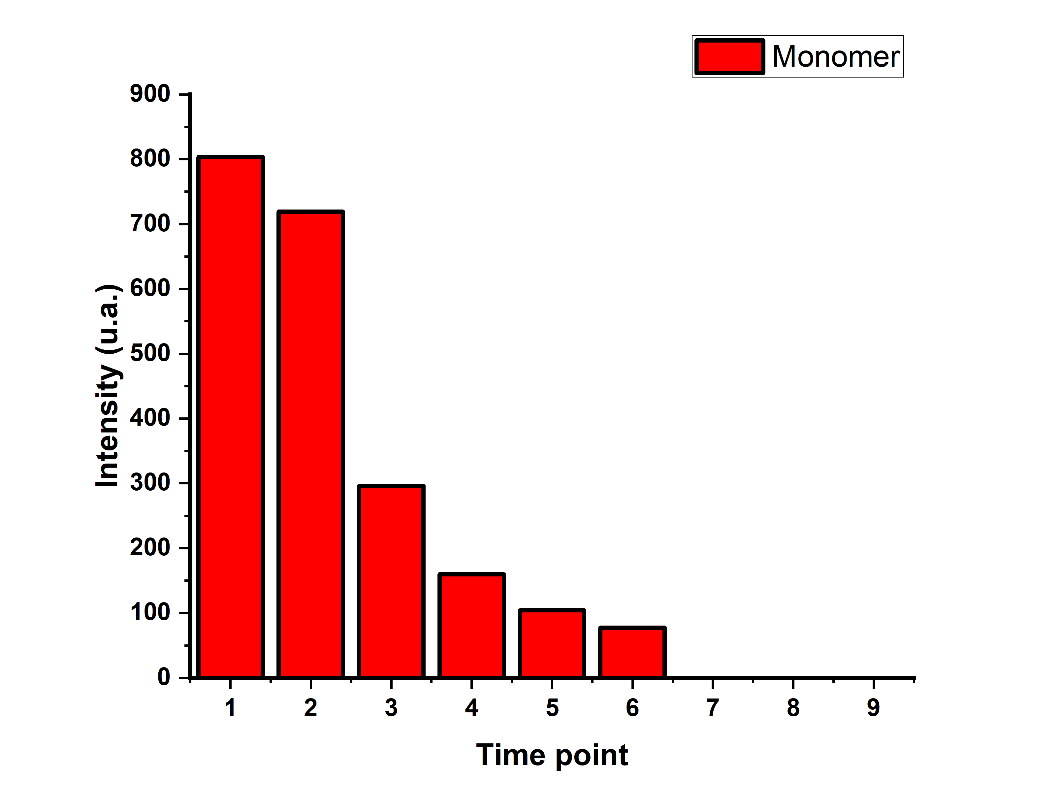


**Figure 5S**: Abundance of the insulin monomeric species at the different time points: 0 min (time point 1) – 45 min (time point 2) – 90 min (time point 3) – 135 min (time point 4) – 195 min (time point 5) – 255 min (time point 6) – 375 min (time point 7) – 495 min (time point 8) - 24 hours (time point 9).


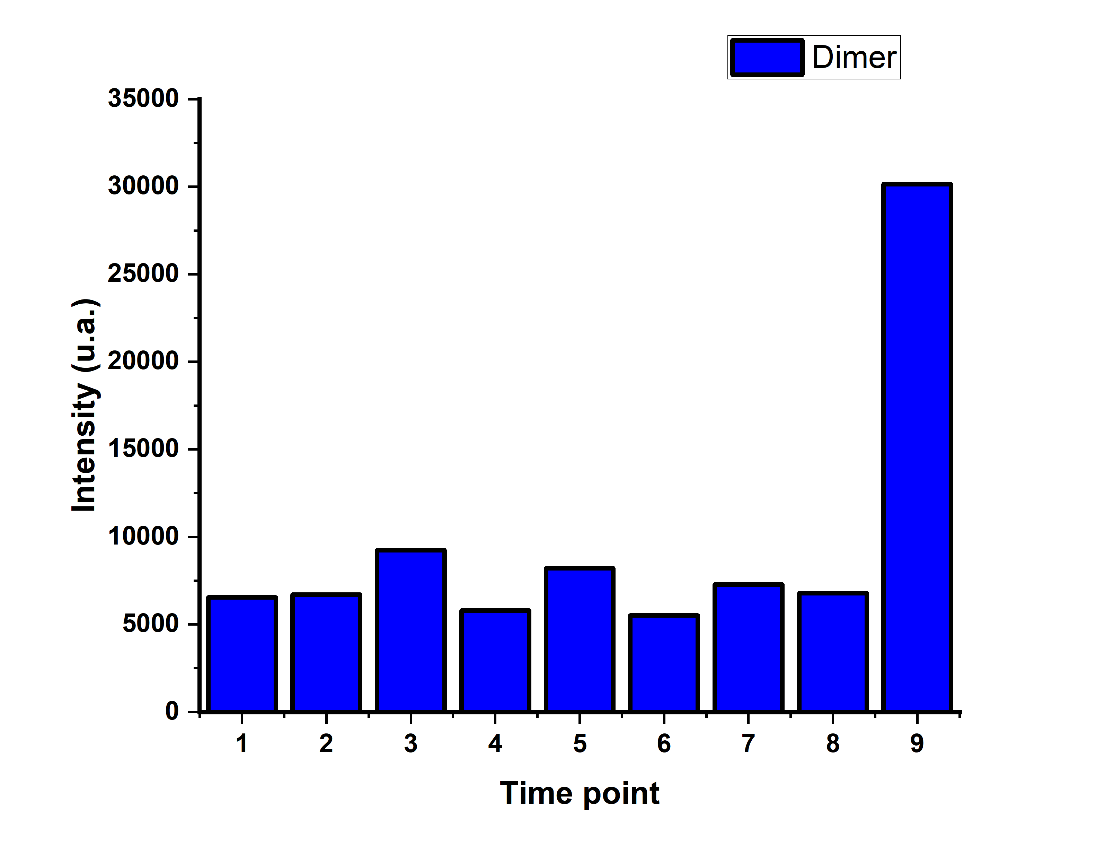


**Figure 6S**: Abundance of the insulin dimeric species at the different time points: 0 min (time point 1) – 45 min (time point 2) – 90 min (time point 3) – 135 min (time point 4) – 195 min (time point 5) – 255 min (time point 6) – 375 min (time point 7) – 495 min (time point 8) - 24 hours (time point 9).

The crosslink reaction induced by the PICUP does not allow to detect the presence of oligomers of a higher order than the dimer, due to the low concentration of these species and a poor sensitivity of the dye. Furthermore, given that the crosslink reaction depends on the stability of the peptide radical which is formed on the protein, not all amino acids have the same reactivity. Some studies have shown that among the 20 natural amino acids, the probability that the side chains of the amino acids tryptophan and tyrosine are the sites of formation and/or reaction of radicals is the highest, while that of the side chain of alanine is the lowest.(1) This suggests that, depending on the aggregated species present in the solution, tyrosine and tryptophan could not be available for the formation of the radical and, consequently, the formation of oligomers of a higher order than the dimer could be hindered. Our results show that, at time point 9 (24h), a very intense signal of the dimeric species is appreciated, due to the reduced distance in space of the peptide species, which, with the used irradiation time of 1.5 s, form a greater number of dimeric species.

*Reference:*

1. *Bitan, G. Structural Study of Metastable Amyloidogenic Protein Oligomers by Photo‐Induced Cross‐Linking of Unmodified Proteins. in Methods in Enzymology vol. 413 217–236 (Academic Press, 2006).*
